# Digital Surface-Enhanced Raman Spectroscopy–Lateral Flow Test Dipstick: Ultrasensitive, Rapid Virus Quantification in Environmental Dust

**DOI:** 10.1021/acs.est.3c10311

**Published:** 2024-03-07

**Authors:** Wei Wang, Sonali Srivastava, Aditya Garg, Chuan Xiao, Seth Hawks, Jin Pan, Nisha Duggal, Gabriel Isaacman-VanWertz, Wei Zhou, Linsey C. Marr, Peter J. Vikesland

**Affiliations:** † Department of Civil and Environmental Engineering, Virginia Tech, Blacksburg, Virginia 24061, United States; ‡ Virginia Tech Institute of Critical Technology and Applied Science (ICTAS) Sustainable Nanotechnology Center (VTSuN), Blacksburg, Virginia 24061, United States; § Department of Electrical and Computer Engineering, Virginia Tech, Blacksburg, Virginia 24061, United States; ∥ Department of Biomedical Sciences and Pathobiology, Virginia Tech, Blacksburg, Virginia 24061, United States

**Keywords:** surface-enhanced Raman spectroscopy, digital analysis, lateral flow test, SARS-CoV-2 detection, indoor dust

## Abstract

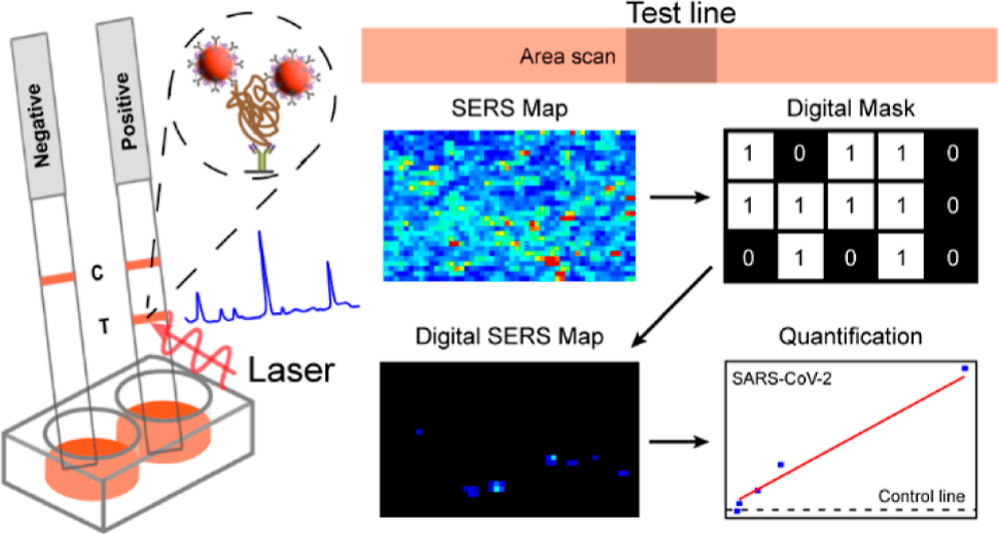

This study introduces a novel surface-enhanced Raman spectroscopy (SERS)-based lateral flow test (LFT) dipstick that integrates digital analysis for highly sensitive and rapid viral quantification. The SERS–LFT dipsticks, incorporating gold–silver core–shell nanoparticle probes, enable pixel-based digital analysis of large-area SERS scans. Such an approach enables ultralow-level detection of viruses that readily distinguishes positive signals from background noise at the pixel level. The developed digital SERS–LFTs demonstrate limits of detection (LODs) of 180 fg for SARS-CoV-2 spike protein, 120 fg for nucleocapsid protein, and 7 plaque forming units for intact virus, all within <30 min. Importantly, digital SERS–LFT methods maintain their robustness and their LODs in the presence of indoor dust, thus underscoring their potential for accurate and reliable virus diagnosis and quantification in real-world environmental settings.

## Abstract 2

Digital analysis of SERS data at the lateral flow test line enhances sensitivity and enables precise and reliable virus quantification in environmental dust.

## Introduction

The persistent threat of viral respiratory diseases is a pressing challenge for regional and global public health.^1−4^ Achieving efficient point-of-care (POC) viral diagnosis for both clinical (i.e., nasal and throat) and environmental (i.e., water and air) samples is crucial for identifying infected individuals, tracing viral transmission pathways, and ultimately implementing effective control strategies.^5−7^ Lateral flow test (LFT)-based detection schemes are globally used for personal diagnosis due to their portability and cost-effectiveness.^8^ Notably, during the COVID-19 pandemic, SARS-CoV-2 antigen test kits, a type of LFT, played a significant role in personalized testing.^9−11^ Commercial LFT kits, generally using antibody-functionalized gold nanoparticles (AuNPs) to indicate the presence of target antigen through visual color changes, can offer rapid, low-cost on-site testing.^12^ However, such LFTs only deliver binary (positive/negative) results and lack the capacity to quantify viral loads.^13^

To address this limitation, surface-enhanced Raman spectroscopy (SERS) has emerged as a novel readout method for LFTs with enhanced capacity for viral quantification.^14,15^ The SERS–LFT approach employs AuNPs prefunctionalized with both antibodies and Raman reporter molecules.^14^ Dual functionalization enables specific interactions between the viral target and the SERS probes, thus facilitating quantification through SERS analysis of the test line.^15^ SERS–LFTs, capable of detecting specific antibodies, viral proteins, or intact viruses,^16−22^ have shown great potential, especially when interrogated using a portable Raman spectrometer for rapid, on-site analysis via single-point or multipoint scanning.^21,23,24^ The limit of detection (LOD) for the SERS–LFT strips is determined by establishing the correlation between the SERS intensity and viral concentration. To minimize data variability from AuNP diffusion on the strip during data point collection, researchers have performed area scans across the test line to produce SERS maps.^25,26^ In these efforts, quantification based on the average intensity across the scanned area is reliable and robust. Nevertheless, conventional quantification methods based on single-point or average intensities are challenging at low and environmentally relevant viral concentrations. This challenge reflects the limited number of captured SERS probes on the test line that result in relatively high levels of background and spatially disparate positive SERS signals across the scan area. A new approach capable of filtering out background data that enables accurate viral quantification at low concentrations is needed.

Recently, Brolo et al. introduced a pixel-based digital analysis approach for interrogation of SERS area scans to achieve single-molecule detection.^27−29^ This technique has subsequently been extended to quantify low concentrations of viruses via SERS-based sandwich immunoassays.^30,31^ In digital SERS analysis, a predefined threshold is established to filter out background pixels from the scanned area, while positive pixels originating from SERS hotspots are retained.^29^ Pixels with intensities below the threshold are background and excluded from further analysis, while those exceeding the threshold reflect the specific localized capture of the SERS probes. A calibration curve is generated by plotting either the number of digital counts or the digital counts multiplied by the measured Raman intensities against the target concentration.^30−32^ In comparison to the average intensity-based quantification method, digitalization allows for direct visualization of SERS probe capture at the individual pixel level, thus enabling ultrasensitive quantification. Despite these considerable achievements, digital SERS analysis for virus detection has primarily found application in fixed substrate-based sandwich immunoassays, which usually take hours^31^ and has yet to be integrated into LFT systems for rapid detection.

**Figure 1:**
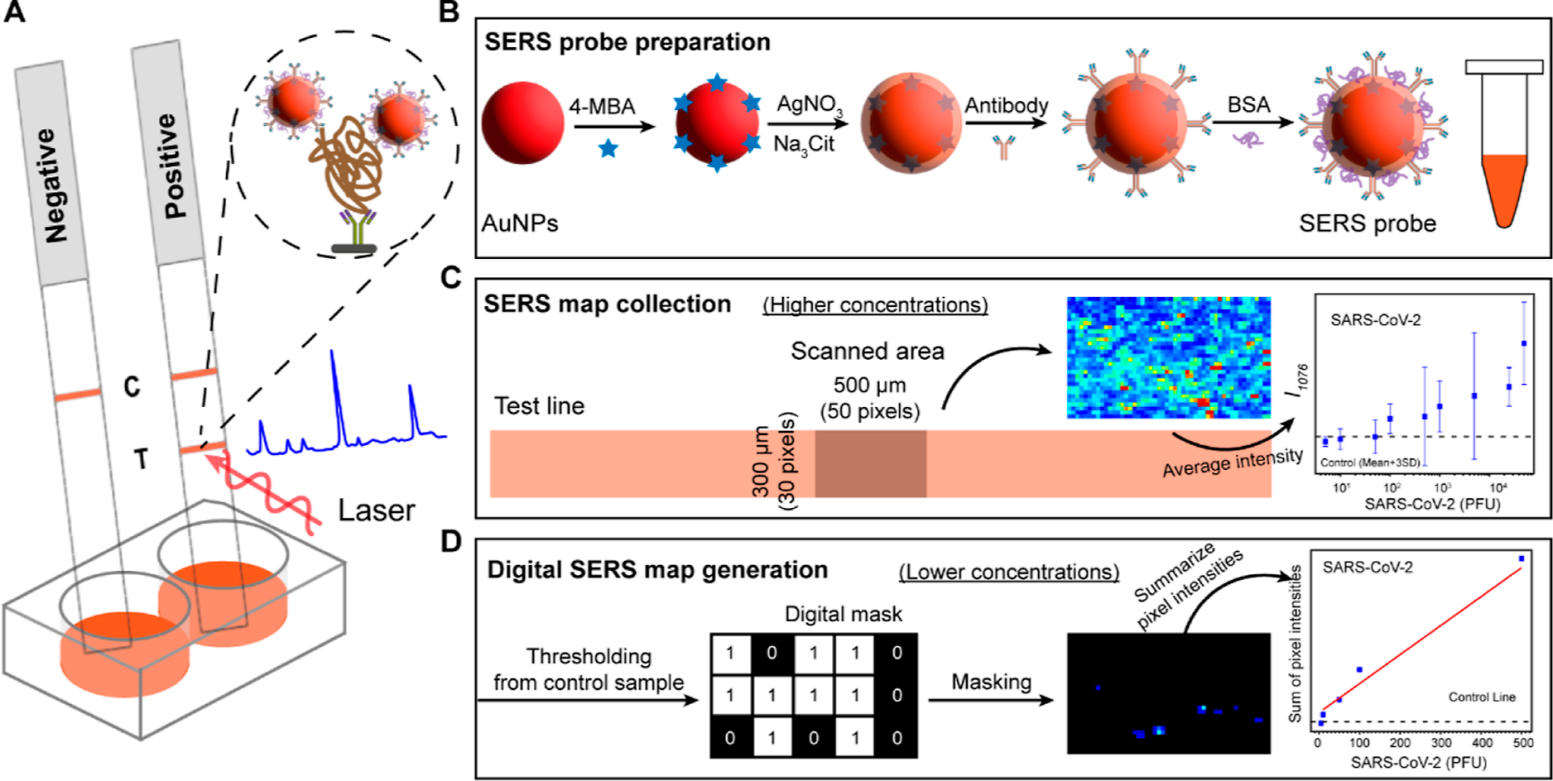
Illustrative overview of the SERS–LFT dipstick for rapid SARS-CoV-2 detection. (A) SERS–LFT assay setup, involving sample mixing with an antibody-functionalized SERS probe and running buffer within a 96-well plate, followed by SERS spectra collection at the test line; (B) process of constructing the SERS probe, entailing the functionalization of the core–shell nanoparticle with Raman reporter and capture antibody; (C) 2D SERS mapping utilized for viral quantification at higher concentrations using average intensity measurements; and (D) 2D digital SERS mapping employed for quantifying lower viral concentrations based on the summation of pixel intensities.

In this study, we developed a SERS-based lateral flow dipstick, coupled with digital SERS analysis, for rapid and ultrasensitive quantification of SARS-CoV-2 (Figure 1). To enhance the signal intensity, we utilized core–shell nanoparticle SERS probes. We initially optimized the lateral flow dipsticks for SARS-CoV-2 spike and nucleocapsid protein detection and then used these optimized dipsticks for intact virus quantification. Subsequently, the dipsticks were used to analyze indoor dust samples. The exceptional sensitivity, portability, cost-effectiveness, and robustness of dipsticks against dust render them well-suited for virus monitoring in the environment, especially in resource-limited regions.

## Experimental Section

### Materials

Gold(III) chloride trihydrate (HAuCl_4_·3H_2_O, ≥99.9%), sodium citrate tribasic dihydrate (Na_3_Cit·3H_2_O, ≥99%), silver nitrate (AgNO_3_, ≥99%), 4-mercapto benzoic acid (4-MBA, ≥95%), ethanol, borax buffer (pH = 9), phosphate-buffered saline (PBS, pH = 7.4), bovine serum albumin (BSA), potassium carbonate (K_2_CO_3_, ≥99%), cellulose fiber sample pad strips (CFSP203000), HI-Flow plus HF135 membrane card (NC membrane attached to a laminated card), and anti-human IgG (Fc specific) antibody produced from goat (I2136) were purchased from Sigma-Aldrich (St. Louis, MO, USA). The SARS-CoV-2 spike protein (S1 subunit) (40591-V06H), SARS-CoV-2 (2019-nCoV) nucleocapsid-His recombinant protein (40588-V08B), SARS-CoV-2 spike antibody (chimeric mAb, 40150-D001), SARS-CoV-2 nucleocapsid antibody (mouse mAb, 40143-MM08 (M08), rabbit mAb, (40143-R004) (R04), rabbit mAb (40143-R001) (R01), and human angiotensin-converting enzyme 2 (ACE2, 10108-H05H) were purchased from Sino Biological. SARS-CoV-2 omicron variant (B.1.1.529) was cultured in Vero E6 TMPRSS2/ACE2 cells in Dulbecco’s modified Eagle’s medium (DMEM). Intact SARS-CoV-2 virus was inactivated via UV-C exposure for 15 min. Inactivation was confirmed by the plaque assay. An automated lateral flow reagent dispenser and running buffer were purchased from ClaremontBio. All glassware was washed with aqua-regia (3:1, HCl/HNO_3_) to remove nanoparticles and other contaminants. Deionized (DI) water with a resistivity greater than 18.2 MΩ was used for all experiments.

### Gold Nanoparticle and Core–Shell SERS Probe Preparation

AuNPs were synthesized through a seed-mediated growth approach.^21,33^ Initially, 75 mL of 2.2 mM Na_3_Cit was heated in a flask until it was boiling. Subsequently, an aliquot of 250 μL of a 50 mM HAuCl_4_ solution was added. The mixture was boiled until a soft pink color developed, indicating the presence of AuNP seeds (∼13 nm). To continue the AuNP synthesis process, 50 μL of 60 mM Na_3_Cit and 25 μL of 50 mM HAuCl_4_ solution were successively added to the seed suspension. This process was repeated 10× at 2 min intervals. Once the suspension reached a deep ruby-red color, it was stirred at 90 °C for 30 min and then further stirred at room temperature for 12 h to allow complete growth of the AuNPs.

The AuNPs were surface-functionalized with the Raman responsive molecule 4-MBA to yield intense SERS signals. Specifically, 20 μL of 10 mM 4-MBA was added to 1 mL of the as-synthesized AuNP suspension, and the mixture was stirred for 10 min at room temperature. To remove excess 4-MBA, the suspension was centrifuged at 7970*g* for 3 min, and then the pellet was resuspended in 2 mL of DI water. To prevent potential surface obstruction by 4-MBA during antibody functionalization, a silver shell was introduced onto the AuNP probe to form a core–shell structure (Figure 1B). To produce these core–shell nanoparticles, 50 μL of 38.8 mM Na_3_Cit and 50 μL of 8 mM AgNO_3_ were added to 8 mL of AuNP suspension.^34^ This process was repeated 4× at 1 min intervals. The suspension was boiled for another 15 min and then cooled to room temperature. The final core–shell SERS probe (Au^4-MBA^@AgNPs) exhibited an orange color.

### Finite-Domain Time-Difference Simulation

FDTD simulation was used to calculate the electric field of the core–shell SERS probe and to show the enhancement of the Raman signal. Optical constants for Au and Ag were taken from Johnson and Christy.^35^ The refractive index of the interior gap containing 4-MBA was set at 1.0, in accordance with the value reported in the literature.^31^ For the core–shell Au^4-MBA^@AgNP structure, the radius of the core and the thickness of the shell were determined to be 18.2 and 4.7 nm, respectively, based on particle sizes determined via transmission electron microscopy (TEM) image analysis. A perfectly matched boundary layer condition was used in the *x*, *y*, and *z* directions to absorb outgoing waves and to prevent reflections from the simulation domain’s boundaries.

### Capture Antibody-Functionalized Core–Shell SERS Probe Preparation

To prepare the final antibody-functionalized SERS probes, the pH of the Au^4-MBA^@AgNP suspension was controlled between 6.5 and 7.5 by adding 6 μL of 0.2 M K_2_CO_3_ to 1 mL of Au^4-MBA^@AgNP suspension. Subsequently, 1 μL of capture antibody was added to the SERS probe suspension, and the mixture was stirred for 45 min. Antispike antibody (40150-D001, 1 mg/mL) was used as a capture antibody for spike protein detection, while antinucleocapsid antibody (40143-R001, 0.1 mg/mL) was used for the detection of both nucleocapsid protein and intact SARS-CoV-2 virus. Unreacted sites on Au^4-MBA^@AgNP probes were blocked by adding 10 μL of 0.1% BSA and stirring for another 30 min to prevent nonspecific binding. Finally, the suspension was centrifuged at 3540*g* for 10 min to remove excess chemicals and unbound antibodies. The pellet was resuspended in 100 μL of 1 mM borax buffer and stored at 4 °C until further use.

### Dipstick SERS–LFT Strip Preparation and Assay Operation

To simplify the traditional LFT, we used a dipstick setup (Figure 1A). The dipstick was produced by assembling an NC membrane and an absorbent pad. The control and test lines were generated by dispensing 1 mg/mL of antihuman IgG and the corresponding detection recognition elements on the NC membrane at a rate of 0.8 μL/cm, respectively, using a ClaremontBio Automated lateral flow reagent dispenser. ACE2 (0.3 mg/mL) and nucleocapsid antibody (40143-MM08, 0.2 mg/mL) were optimized as effective recognition elements for spike and nucleocapsid proteins, respectively, to achieve a high capture efficiency while minimizing nonspecific binding. The dispensed NC membrane was allowed to air-dry for a minimum of 30 min at room temperature before use. Subsequently, the absorbent pad was attached to the top of the NC membrane with a 2 mm overlap. The final 2.5 mm wide strips were obtained by using a cutter.

The dipstick strip was placed into the well of a 96-well plate for sample detection (Figure 1A). During initial assay development, SARS-CoV-2 spike protein and nucleocapsid protein were employed as targets. In this procedure, 10 μL of antibody-functionalized Au^4-MBA^@AgNP SERS probe was mixed with 10 μL of target protein for 10 min and added to 30 μL of running buffer in a well of a 96-well plate. The dipstick strip was then inserted into the well for sample detection. The SERS probe migrated upward within 15–20 min. Following this diffusion period, 10 μL of PBS was added to the well to minimize nonspecific binding. For each concentration, triplicate strips were tested.

SERS spectra at the test line were acquired using a WITec alpha500R Raman spectrometer (WITec GmbH, Ulm, Germany, spectral resolution ≈3.5 cm^–1^) with a Peltier cooled charge-coupled device, 785 nm laser, and 300 grooves per mm grating. The SERS signal from the test line of each strip was collected via large-area scanning (Figure 1C). The test line had dimensions of 2.5 mm in length and 300 μm in width. A total of 1500 spectra (50 × 30, *X* × *Y*) were acquired within the middle of the strip across a 500 μm × 300 μm area, thus spanning the entire width of the test line. Measurements were obtained with a 10× confocal microscope objective lens, a laser power of 50 mW, and an integration time of 0.1 s for each point. The raw spectral data were preprocessed using the WITec instrument-embedded software (Project Five) for cosmic ray removal, Savitzky–Golay smoothing, and baseline subtraction. Subsequently, SERS maps were generated based on the peak intensities of the Raman reporter 4-MBA at 1076 cm^–1^. Average peak intensities at 1076 cm^–1^ across the scanned area were calculated for quantification.

Following assay optimization, the strip was used to detect the intact UV-inactivated SARS-CoV-2 virus. To prevent SERS probe aggregation in the viral culture medium, 10 μL of the virus sample was mixed with 30 μL of running buffer and 10 μL of the antibody functionalized SERS probe. The LODs for spike protein, nucleocapsid protein, and SARS-CoV-2 virus using these SERS–LFT strips were determined across a concentration range of 0–500 ng, 0–1 μg, and 0–5000 PFU, respectively.

### SARS-CoV-2 Detection in Indoor Dust Samples

To verify the performance of our SERS–LFT for potential environmental applications, we spiked the SARS-CoV-2 virus into collected indoor dust samples. Indoor environments contain airborne particulate matter at concentrations up to 100 μg/m^3^. Dust is a mixture of particulate matter from outdoor air plus that arising from indoor sources, including biological matter (e.g., pollen, dead skin, bacteria, and viruses).^36−38^ Dust was collected by vacuuming an HVAC filter acquired from a local school. The vacuumed dust was then suspended in ultrapure water at 5 mg/mL. The dust stock was diluted to 0.5 mg/mL, which roughly corresponds to the maximum amount of dust expected in a 5 m^3^ sample of air within an indoor environment and eluted into 1 mL of solvent. This volume of air is what a person inhales over several hours and what might be collected with a sampler designed to detect viruses in the air. Different concentrations of UV-inactivated SARS-CoV-2 were spiked into the dust suspensions to achieve final concentrations of 0–2500 PFU. SARS-CoV-2 detection in dust samples was performed using a protocol described previously.

### Digital SERS-Enabled Quantification

We utilized a pixel-based digital SERS analysis approach to achieve accurate viral quantification over a broad concentration range. For digital SERS analysis, the SERS map was converted into a binary format (1 = positive or 0 = negative) based on whether a given pixel’s intensity exceeded a predefined threshold. The threshold was set based on the SERS data collected from the negative control sample (sample without viral target), as discussed in detail later. Pixels with intensities below the threshold were set to 0, while those surpassing the threshold were set to 1. The digitized map was then combined with the original SERS map to generate a digital SERS map. Subsequently, the sum of intensities above the threshold for each concentration was used for quantification.

## Results and Discussion

### Core–Shell SERS Probe Characterization

**Figure 2:**
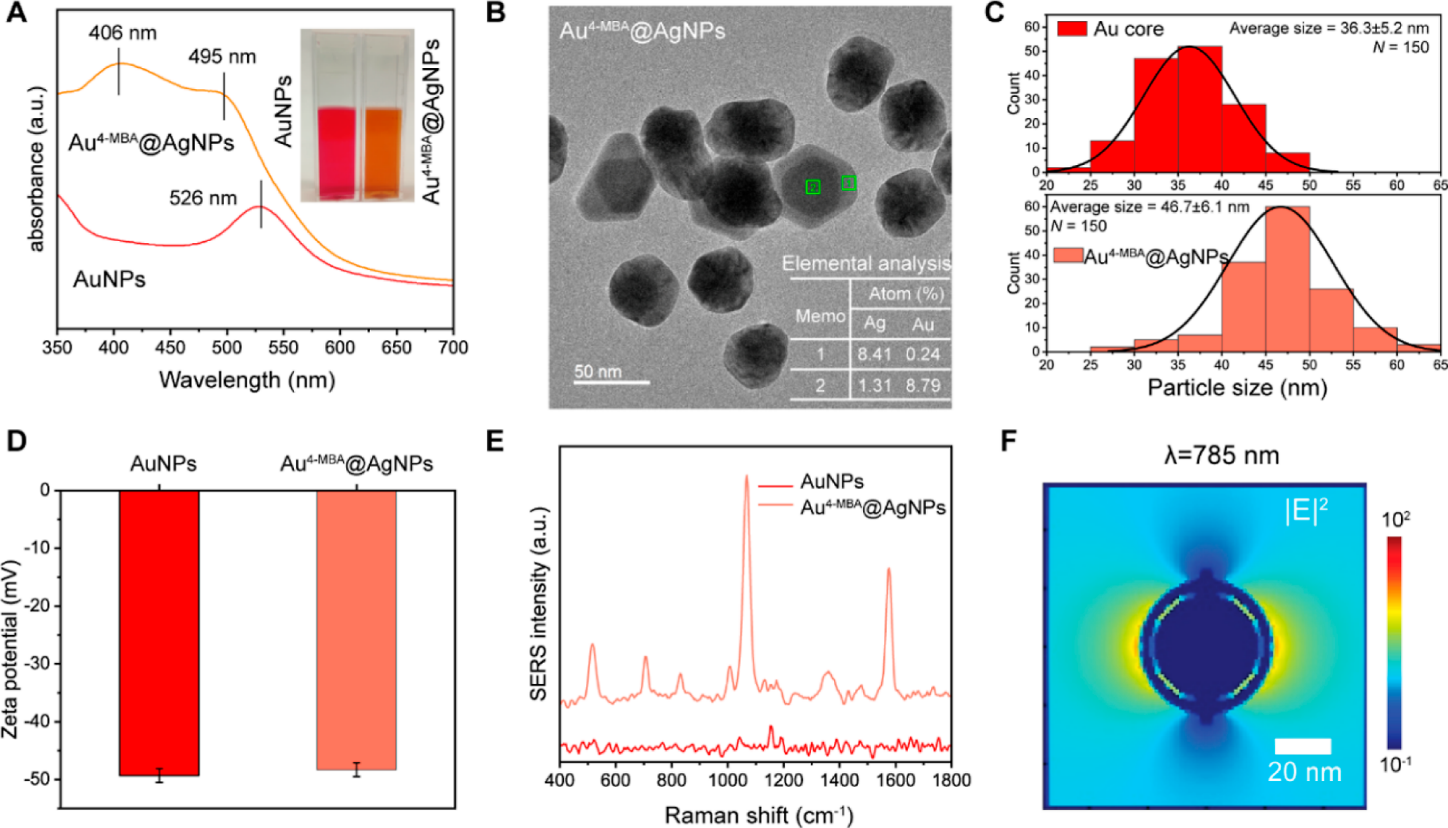
Characterization of AuNPs and core–shell Au^4-MBA^@AgNPs. (A) UV–visible spectra. (B) TEM image of the core–shell Au^4-MBA^@AgNPs. The table shows the EDS elemental analysis from core (rectangle 2) and shell (rectangle 1). (C) Analysis of the average particle sizes of the Au core and the Au^4-MBA^@AgNPs, calculated from TEM images (*N* = 150). (D) Zeta potential measurements. (E) Comparative SERS spectra of 4-MBA-functionalized AuNPs and core–shell Au^4-MBA^@AgNPs. (F) FDTD-calculated electric field intensity |*E*|^2^ distribution map at 785 nm.

We prepared Au^4-MBA^@AgNP SERS probes with a core–shell structure to prevent potential competition among adsorption sites during Raman reporter modification and capture antibody functionalization. The Raman reporter 4-MBA was functionalized to an AuNP surface and then enveloped by a thin Ag shell. The Ag shell serves a dual role as it enhances the SERS signal and provides binding sites for antibody functionalization (Figure 1B). Figure 2A shows that the as-produced AuNPs exhibited a distinct localized surface plasmon resonance (LSPR) peak at 526 nm, and the suspension exhibits a red-pink color. Following 4-MBA functionalization and Ag shell formation, the resulting Au^4-MBA^@AgNPs had an orange color and exhibited two LSPR peaks at 495 and 406 nm, corresponding to the Au core and Ag shell, respectively. The formation of this core–shell structure was confirmed through high-resolution TEM imaging. The TEM image in Figure 2B shows the distinguishable contrast between the dark Au core and the lighter Ag shell, which can be attributed to the lower atomic weight of Ag. Elemental analysis supports this observation, with a higher percentage of Ag in the shell and a higher percentage of Au in the core. The average diameters of the Au core and the Au^4-MBA^@AgNPs determined by TEM were 36.3 ± 5.2 and 46.7 ± 5.1 nm, respectively, indicating an average Ag shell thickness of approximately 5.2 nm (Figure 2C). The zeta potentials of the AuNPs and Au^4-MBA^@AgNPs were −49.3 and −48.2 mV, respectively (Figure 2D). This result indicates that formation of the Ag shell had minimal impact on the electronegativity of the nanoparticles.

The SERS performance of the probes was evaluated by using a dilute colloidal suspension. This condition enables nanoparticle dispersion, representing limited nanoparticle capture on the test line when the target concentration is low. In this condition, 4-MBA exhibits a much stronger SERS intensity on core–shell Au^4-MBA^@AgNPs compared to that on AuNPs (Figure 2E). The spatial distribution of the calculated electric field intensity (|*E*|^2^) indicate strong local electric fields in the gap between the Au core and the Ag shell when excited with a wavelength of 785 nm, as confirmed by finite-domain time-difference (FDTD) simulation (Figure 2F). The enhanced electric field in the gap significantly amplifies the Raman intensity of 4-MBA, leading to an observed higher SERS intensity. To enhance the SERS performance, we optimized the thickness of the shell by varying the AgNO_3_ dosage. As the dosage was increased, a more prominent Ag LSPR peak was observed (Figure S1A). Upon reaching an AgNO_3_ dosage of 200 μL, 4-MBA exhibited the highest enhancement (Figure S1B,C). Consequently, we maintained this specific condition for Au^4-MBA^@AgNP SERS probe synthesis.

### SARS-CoV-2 Protein-Enabled SERS–LFT Construction

We initially developed the SERS–LFT using a commercially available SARS-CoV-2 spike protein. Nonspecific binding between the detection antibody at the test line and the nanoparticle capture antibody is a significant challenge for LFTs.^39^ To address this issue, we selected ACE2, a known receptor for the receptor-binding domain of SARS-CoV-2 spike protein,^40^ which has reportedly lower nonspecific binding relative to antibodies,^39^ as the recognition element at the test line. We initially optimized the ACE2 concentration with a simple dot-blot assay, in which ACE2 was directly drop-cast onto the NC membrane. Figure S2 shows that ACE2 at a concentration of 1 mg/mL yielded the highest SERS intensity when interacting with spike protein and SERS probe while also effectively reducing nonspecific binding. However, when we used a line dispenser for practical LFT implementation, we found that further reduction to 0.3 mg/mL best reduced nonspecific binding. This lower concentration reflects greater ACE2 dispersion during line printing.

**Figure 3:**
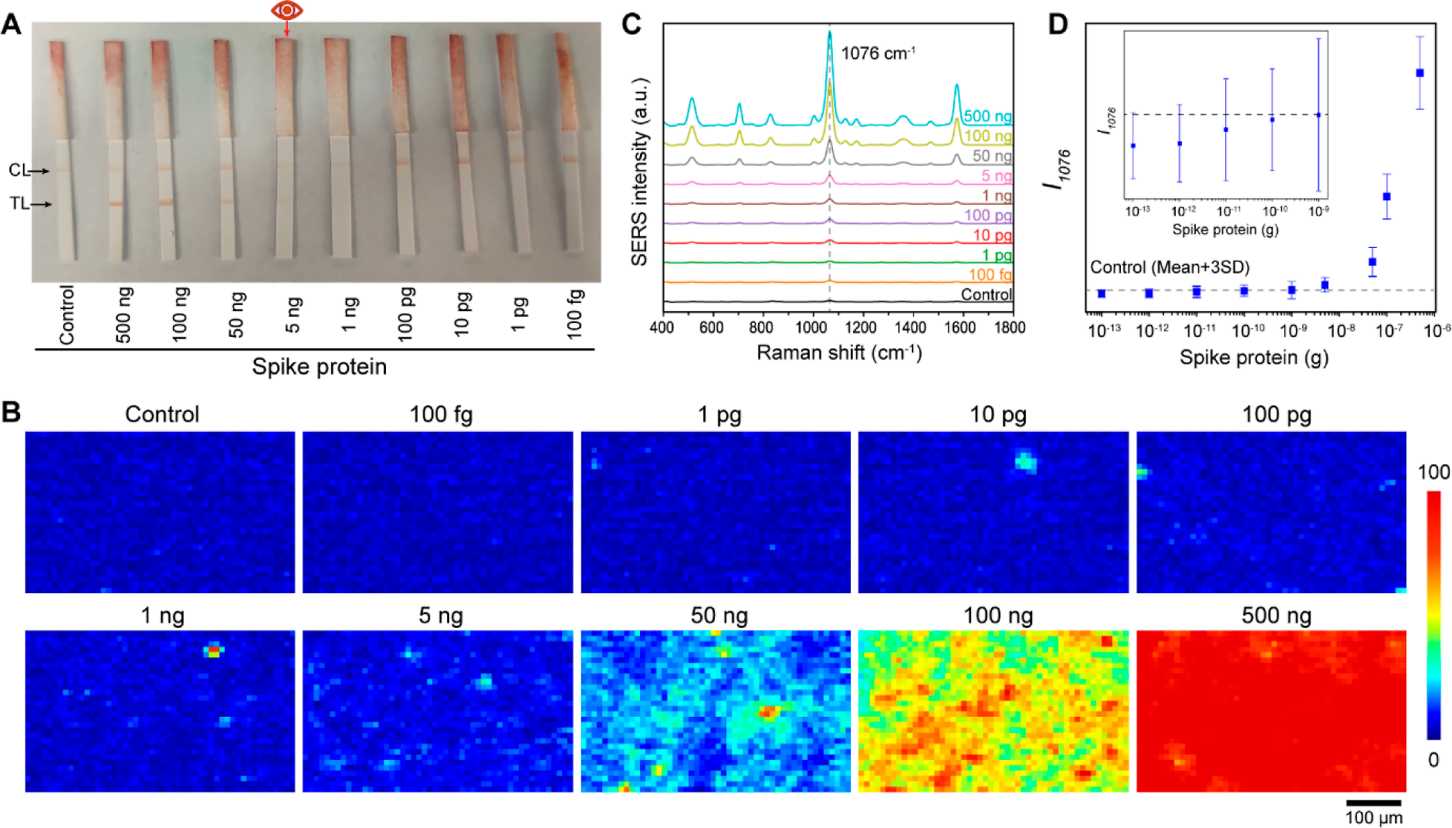
SERS–LFT applications for SARS-CoV-2 spike protein detection. (A) Visual photographic representation of the SERS–LFT strips post-detection at varying spike protein amounts; (B) measured 2D SERS maps and (C) average SERS spectra from 2D scanning over the test line areas, based on the peak intensity at 1076 cm^–1^ (*I*_1076_) for samples at varying spike protein amounts; and (D) comparative analysis of *I*_1076_ for samples at varying spike protein amounts together with the control sample. The error bars represent the standard deviations obtained from the area scans of each sample.

We applied the optimized SERS–LFT strips for spike protein quantification. Figure 3A displays a photograph of the SERS-LFT strips following the application of different amounts of spike protein. The lowest concentration for spike protein detection was determined to be 5 ng based on the colorimetric readout, as indicated by the very faint color at the test line. This value is similar to the value in previously reported for ACE2-based colorimetric LFTs for spike protein detection.^39^ To check the LOD based on the SERS readout, Raman spectra were acquired from the test line over a 500 μm × 300 μm area (1500 points; 50 × 30 points). This spatial area was chosen as it aligned with the width of the test line (300 μm) and provided a representative area in its center. SERS maps were generated across the scanned area based on the peak intensity at 1076 cm^–1^, the most intense peak for SERS reporter 4-MBA (Figure 3B). The SERS intensity increases with an increased spike protein concentration, indicating enhanced nanoparticle capture at the test line. Afterward, we calculated the average peak intensity across the entire area. The averaged results show that when the spike protein exceeds 1 ng, the intensity is distinguishable relative to that of the control sample (Figure 3C,D).

Similar SERS–LFT strips were developed for nucleocapsid protein detection. As emphasized previously, antibody selection is crucial toward enabling target detection and mitigating nonspecific binding. Accordingly, we evaluated six antibody combinations involving capture antibody on the SERS probe and detection antibody on the test line, all derived from three distinct nucleocapsid protein antibodies.^41^ We expected to identify the optimal antibody pair that provides a robust colorimetric signal in the presence of the target while concurrently minimizing nonspecific binding in its absence. Figure S3 clearly shows that the most effective combination was obtained by using R01 as the capture antibody on the SERS probe and M08 as the detection antibody on the test line. Using this combination, we employed the SERS–LFT strip for nucleocapsid protein quantification (Figure S4). The colorimetric LOD was visually determined to be 1 ng, a value comparable to that of the spike protein. The SERS-based readout shows there is a significant intensity increase in the spectrum at 10 pg for nucleocapsid protein based on the average SERS intensity across the scanned area (Figure S4D). This value is 2 orders of magnitude more sensitive than the colorimetric readout.

Despite the improvement in the LOD based on the average SERS intensity, we recognized that this improvement may be insufficient for sensitive environmental analysis. To potentially address this limitation, further analysis of individual pixels potentially offers increased sample quantitation. As shown in Figure 3B, when the concentration of spike protein is lower than 1 ng, certain pixels on the SERS map exhibit notably higher intensities despite the observed similarity in spatially averaged intensities. This observation is supported by Figure 3D, where the upper end of the error bar surpasses the control value. Analyzing individual pixel intensities could potentially lead to further reductions in the LOD.

### Digital SERS-Enabled Quantification

**Figure 4:**
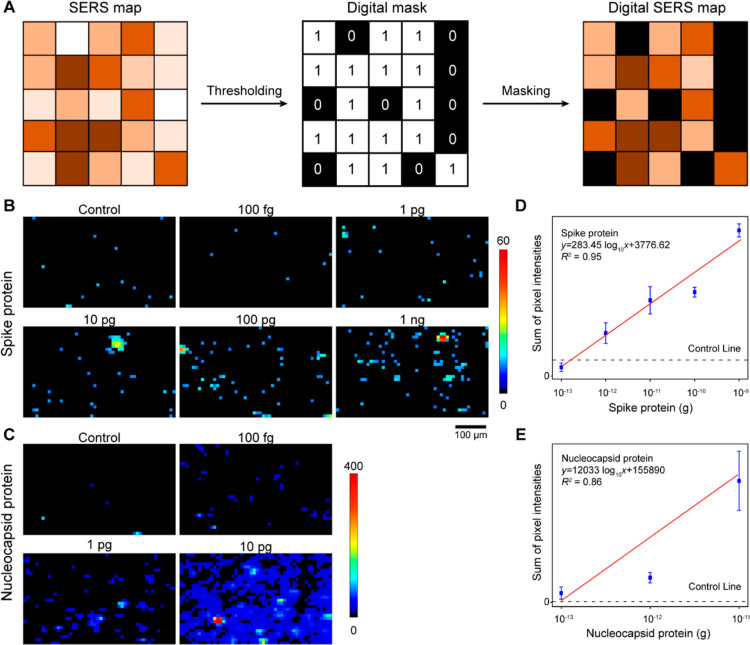
Digital SERS for quantitative analysis of spike protein and nucleocapsid protein amounts. (A) Conceptual diagram of digital SERS methodology; (B) digital SERS maps for spike protein amounts from 100 fg to 1 ng; (C) digital SERS maps for nucleocapsid protein amounts from 100 fg to 10 pg; and correlation of (D) spike protein concentration and (E) nucleocapsid protein amount with cumulative pixel intensities from digital SERS maps. The control line reflects the cumulative pixel intensities of the control strip. The error bars are the standard deviations from triplicate test strips.

Digital SERS was implemented to ensure accurate signal quantification over a lower target concentration range. Practically, when the target concentration is extremely low, only a limited number of SERS probes can carry the target and capture it on the test line, thus leading to spatially heterogeneous SERS intensities. By focusing on the pixels of positive capture, we can decrease the LOD. To achieve this, we employed digital SERS analysis (Figure 4A).^30,31^ In this approach, the SERS map was converted to a binary format by setting a specific threshold. We defined the threshold and calculated the LOD based on the average intensity plus three times the standard deviation (average + 3SD) for the negative control.^42,43^ This threshold selection effectively reduces the background signal arising from nonspecific binding in the control sample and suggests that 99.7% of the pixels in a negative sample should be below this threshold. Pixels with intensities below the threshold were assigned to 0, while those above the threshold were set to 1. The digitized map was then used as a mask and combined with the original SERS map, thus generating a digital SERS map. In this digital SERS map, only pixels with intensities above the threshold are considered. Subsequently, the intensities above the threshold were aggregated and used for quantification. Using pixel-based digital SERS analysis, no false positives or false negatives were observed.

Figure 4B,C displays digital SERS maps for spike and nucleocapsid proteins at low concentration ranges (0–1 ng for spike protein and 0–1 pg for nucleocapsid protein) that are challenging to effectively discern using average SERS intensities. The digital SERS maps reveal that the negative controls are predominantly black, indicating minimal signal with only a few pixels (∼0.3%, as statistically expected) showing intensities above the threshold due to nonspecific binding. When the protein concentration was increased, the pixel count above the threshold also increased, indicating successful capture of the SERS probe in the presence of protein (Figure S5). The different pixel counts and intensities observed for spike and nucleocapsid proteins may be attributed to variations in the affinities of these proteins for their respective antibodies on both SERS probes and test lines. Upon summing the intensities across the maps for triplicate strips (Figure 4D,E), we observed a linear trend between intensity and protein concentration and determined that the SERS–LFT achieved LODs of 180 fg for spike protein and 120 fg for nucleocapsid protein. These values are 3–4 orders of magnitude more sensitive than the colorimetric readout (5 and 1 ng, respectively), thus highlighting the substantial improvement in sensitivity achieved through digital SERS analysis. These LODs are similar to those for other digital SERS-based assays for SARS-CoV-2 protein detection (Table S1). However, given the limited data set presented in Figure 4E, it should be noted that the calculated LOD for nucleocapsid protein may vary on the basis of the calibration curve used.

Selecting an appropriate threshold is a crucial aspect of digital SERS analysis, as it dictates the retention of positive pixels while eliminating background pixels. In this study, we chose a threshold of average intensity plus three times the standard deviation from the control sample.^42^ It should be noted that alternative thresholding methods have been used elsewhere.^30−32^ In this instance, we sought to ensure that our chosen threshold was reasonable and that alterations to this value would not significantly impact the LOD. Accordingly, we generated digital SERS maps and the corresponding sum of intensity plots for the nucleocapsid protein using varying thresholds. As shown in Figure S6, although an increase in the threshold value resulted in removal of greater numbers of “positive” pixels, all of the tested thresholds exhibited a similar detection limit. It should be noted that the threshold values should be reasonable. If we continuously increase the threshold, then useful data from the positive samples may be eliminated, leading to SERS intensity levels that are closer to those of the controls. Previous studies have used the number of digital counts or digital counts multiplied by the Raman intensities for quantification.^30,31^ Given the potential for multiple SERS probes to be captured within each scanned pixel (10 × 10 μm), herein we summed the intensities of the positive pixels.

### Intact SARS-CoV-2 Virus Detection

**Figure 5:**
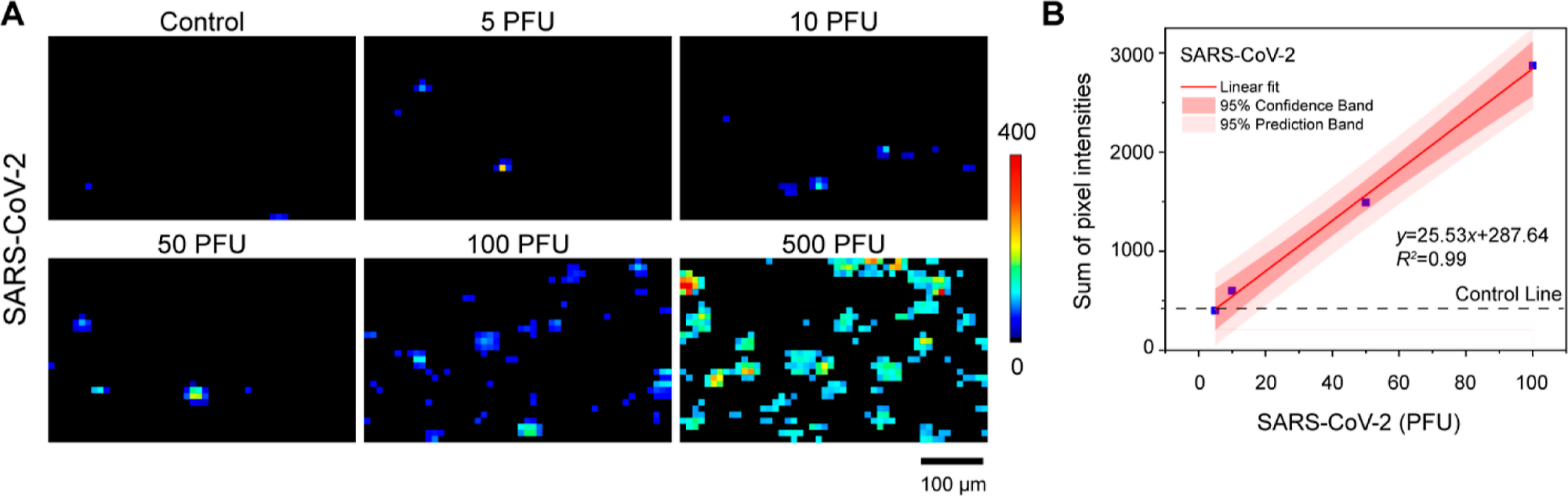
Digital SERS–LFT for quantification of the intact SARS-CoV-2 virus. (A) Digital SERS maps for SARS-CoV-2 ranging from 5 to 500 PFU. (B) Correlation of the SARS-CoV-2 virus amount with cumulative pixel intensities from digital SERS maps.

Following demonstration that the developed digital SERS–LFTs can be successfully deployed for SARS-CoV-2 protein quantification, we applied this method to detect the intact SARS-CoV-2 virus. Active SARS-CoV-2 virus was propagated, suspended in viral transport medium (VTM), quantified using a plaque assay, and then inactivated via UV exposure for biosafety reasons. While applying the strips developed for spike protein detection to the intact SARS-CoV-2 virus, we encountered a significant issue of strong nonspecific binding, even in the control samples. One possible reason for this observation is that substances present in the VTM or originating from cell debris may act as bridge molecules between ACE2 on the test line and the antibody on the SERS probe, thereby leading to nonspecific binding. Such a possibility requires further investigation but was outside the scope of the current study. The strips designed for nucleocapsid protein detection worked well for intact SARS-CoV-2 virus with no nonspecific binding observed (Figures S7 and S8). Accordingly, we used such strips for the detection of the SARS-CoV-2 virus in the following studies. Figure 5 shows a statistically significant correlation (*R*^2^ = 0.99) between virus concentration and the sum of pixel intensity after digital analysis. The experimental data fall within the 95% prediction region, indicating the robustness of the correlation. The LOD was determined to be 7 PFU, a value comparable to that determined for a recent SERS–LFT.^21^ The detection range was 5–2500 PFU by combining digital analysis and average intensity analysis. Compared with single-point or multi-point analyses,^21,23,24^ our use of area scanning and digital analysis mitigates the impact of the spatial heterogeneity of the captured SERS probes and ensures a more robust and reliable output. We also checked the LODs of several commercially available antigen test kits, all of which are based on nucleocapsid recognition, under the same conditions as our SERS–LFT. As shown in Figure S9, each of these kits has a visual detection of ∼100 PFU. These results suggest that our developed digital SERS–LFT is approximately 10× more sensitive than commercial kits.

### SARS-CoV-2 Detection in Indoor Dust

**Figure 6:**
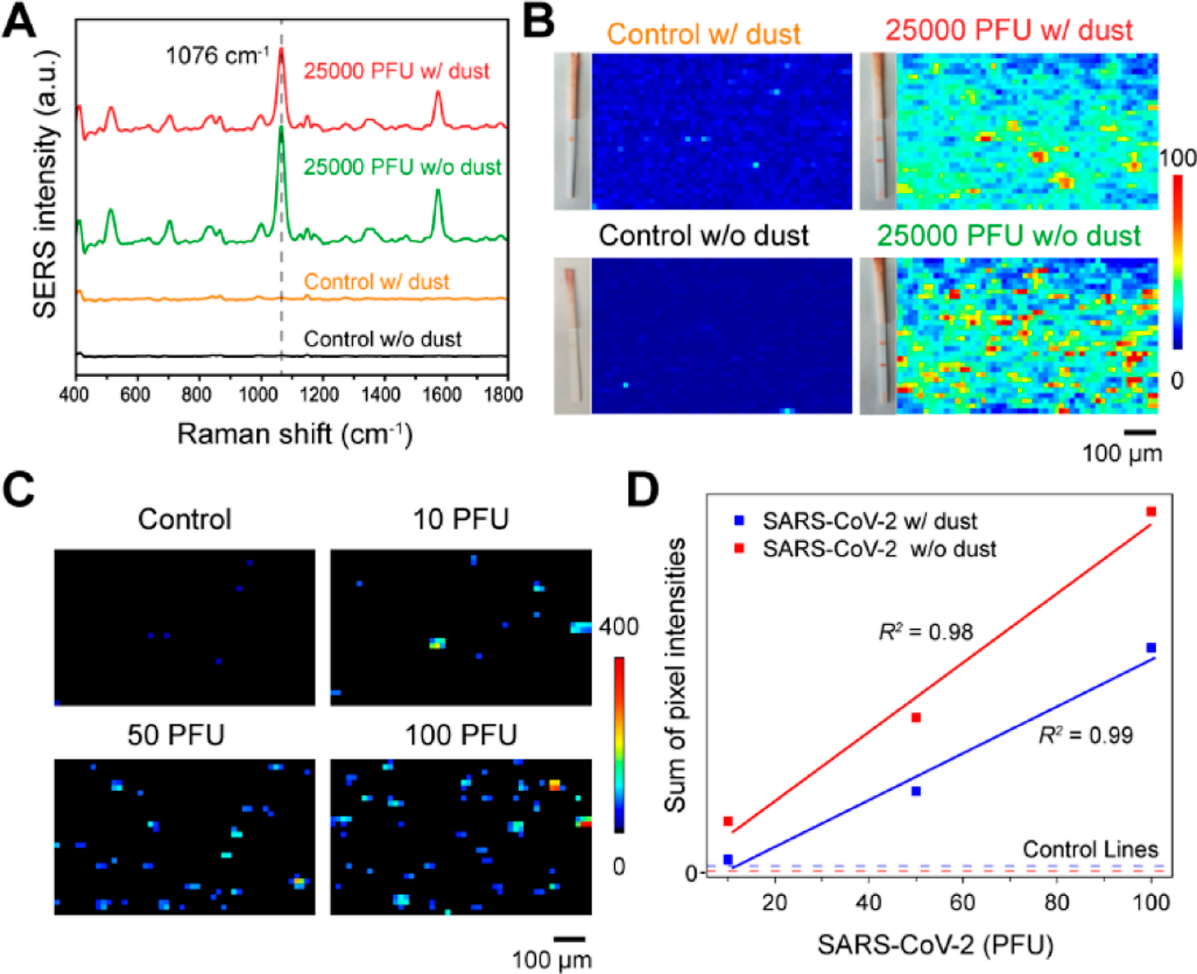
SERS–LFT for the detection of intact SARS-CoV-2 viruses amidst indoor dust. (A) SERS spectra comparison between control and 25,000 PFU virus samples, with and without dust presence. (B) Display of lateral flow strips and corresponding SERS maps for these samples. (C) Digital SERS maps for the SARS-CoV-2 samples with varying PFUs with dust presence. (D) Correlation of the SARS-CoV-2 virus amount with cumulative pixel intensities from digital SERS maps for samples with dust presence.

The detection of airborne SARS-CoV-2 in indoor environments is required to better understand its fate and transport and provide improved opportunities for surveillance. To assess the potential applicability of the developed digital SERS–LFT under such conditions, we spiked SARS-CoV-2 into a suspension containing 0.5 mg/mL of indoor dust in water. This concentration simulates dust collected from 5 m^3^ of air with a background dust concentration of approximately 100 μg/m^3^ that was then eluted in 1 mL of solvent. Figure 6A,B presents the SERS spectra and SERS map of the control sample and the sample containing 25,000 PFU of virus, both in the presence and absence of dust. These spectra demonstrate that dust does not significantly impact the detection of SARS-CoV-2 when the virus concentration is relatively high. However, when the SARS-CoV-2 concentration is low, such as in a negative control with dust present, although no obvious color change was observed on the test line, it exhibited higher SERS intensity compared to the sample without dust. Based on our prior results, we anticipated that applying digital SERS could effectively mitigate such an impact. To validate this hypothesis, we checked the SERS performance of the strips toward SARS-CoV-2 in dust samples (Figure S10). The LOD after digital SERS analysis remains at 10 PFU (Figure 6C,D), matching the LOD obtained without dust. This finding highlights the robustness and reliability of the digital SERS–LFT even in the presence of dust, suggesting its promise for accurate SARS-CoV-2 detection in real-world indoor environments.

### Environmental Implications

This study reports a rapid and ultrasensitive SERS–LFT based on digital analysis for the detection of SARS-CoV-2 proteins and intact viruses and demonstrates its potential applicability for viral quantification in real-world environmental settings. The integration of digital SERS-based analysis with LFT enables both ultrasensitivity and rapidity. Our digital SERS–LFT achieved LODs for SARS-CoV-2 spike protein, nucleocapsid protein, and intact SARS-CoV-2 virus at 180, 120, and 7 PFU in 30 min, respectively. Moreover, the ability of digital SERS–LFT to detect both inactivated viruses and viral proteins ensures its application in a complex dust environment, where the viral viability and protein configurations are not clearly defined. While colorimetric readout-based LFTs have demonstrated quantification capabilities,^44^ SERS-based readout offers better sensitivity since individual SERS nanoprobe can generate robust intensity. The LODs derived from digital SERS analysis in our study are 10× superior to those obtained using commercially available colorimetric readout kits. It has been widely recognized that airborne transmission through aerosols and droplets serves as one of the primary routes for respiratory virus infection, especially in poorly ventilated indoor environments. Despite this, the concentration of SARS-CoV-2 in ambient air is usually very low or undetectable, even in quarantine hospital rooms that contain infected patients.^45^ Effective sampling strategies involving the collection of large air volumes or virus detection in heating, ventilation, and air conditioning (HVAC) systems are often necessary.^46,47^ Given that indoor samples may contain a high density of airborne particulate matter, especially after sample concentration, which could potentially influence nanoparticle stability and color visualization, the application of digital SERS analysis can effectively counter such impacts. Although our digital SERS–LFT may not match the sensitivity of the current standard gene amplification-based SARS-CoV-2 virus detection methods such as quantitative polymerase chain reaction (qPCR) or droplet digital PCR (ddPCR), we envision its use for rapid and accurate virus detection indoors without the need for the tedious sample pretreatment. Moreover, the use of paper-based materials and the advent of hand-held Raman devices enhance its potential cost-effectiveness compared to that of PCR-based methods. This study highlights the potential of digital SERS–LFT methods for rapid monitoring of the transmission of viral respiratory diseases and aids in effective disease control.

## Supporting Information Available

Additional information on SERS probe optimization; antibody optimization, SARS-CoV-2 nucleocapsid protein detection, digital SERS thresholding, intact SARS-CoV-2 virus detection using SERS–LFT and commercial kits, intact SARS-CoV-2 quantification in indoor dust samples, and the performance of other digital SERS-based assays for SARS-CoV-2 detection (PDF)
