# Supplementary material for: Digital Surface-Enhanced Raman Spectroscopy–Lateral Flow Test Dipstick: Ultrasensitive, Rapid Virus Quantification in Environmental Dust
Source: Environ Sci Technol. 2024 Mar 7;58(11):4926–36. doi: 10.1021/acs.est.3c10311 (PMC10956432; doi:10.1021/acs.est.3c10311)
Supplement: Supplementary file 1 — es3c10311_si_001.pdf [file es3c10311_si_001.pdf]

Supporting Information for

Digital SERS-LFT Dipstick: Ultra-sensitive, Rapid Virus Quantification in Environmental Dust

Wei Wang, [1†‡] *Sonali Srivastava,* [1†‡] *Aditya Garg,* [§] *Chuan Xiao,* [§] *Seth Hawks,* [ǂ] *Jin Pan,* [†] *Nisha Duggal,* [ǂ] *Gabriel Isaacman-VanWertz,* [†] *Wei Zhou,* [§] *Linsey C. Marr,* [†‡] *and Peter J. Vikesland* *[†‡]

[†] Department of Civil and Environmental Engineering, Virginia Tech, Blacksburg, Virginia 24061, United States

[‡] Virginia Tech Institute of Critical Technology and Applied Science (ICTAS) Sustainable Nanotechnology Center (VTSuN), Blacksburg, Virginia 24061, United States

[§] Department of Electrical and Computer Engineering, Virginia Tech, Blacksburg, Virginia 24061, United States

[ǂ] Department of Biomedical Sciences and Pathobiology, Virginia Tech, Blacksburg, Virginia 24061, United States

[1] W.W. and S.S. contributed equally to this paper.

*Corresponding Author: Peter J. Vikesland; Email: pvikes@vt.edu.

The Supporting Information contains 6 pages, 10 figures and 1 table.

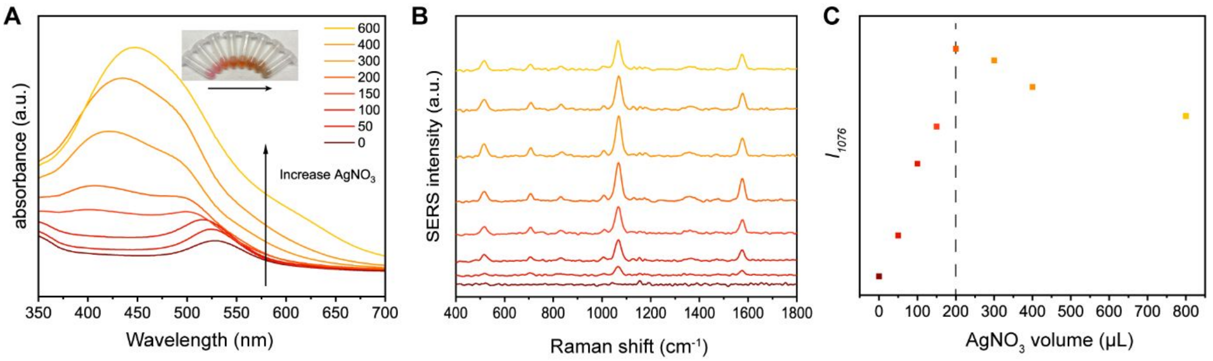

Figure S1. Optimization of shell thickness in core-shell Au$^{4\text{-MBA}}$@AgNPs by varying $AgNO_3$ dosage during synthesis. (A) UV-vis spectra; (B) Average SERS spectra obtained from different Au$^{4\text{-MBA}}$@AgNPs (C) The average peak intensity at 1076 cm^{-1} obtained from different Au$^{4\text{-MBA}}$@AgNPs under different $AgNO_3$ dosage.

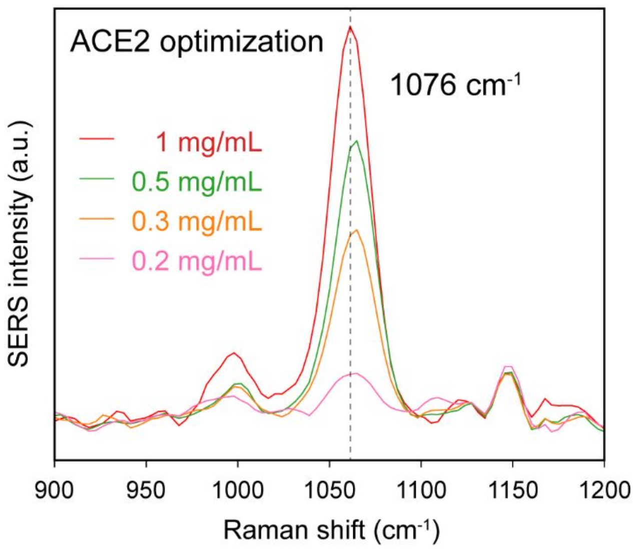

Figure S2. SERS intensity on the test dot during ACE2 concentration optimization using a dot-blot assay.

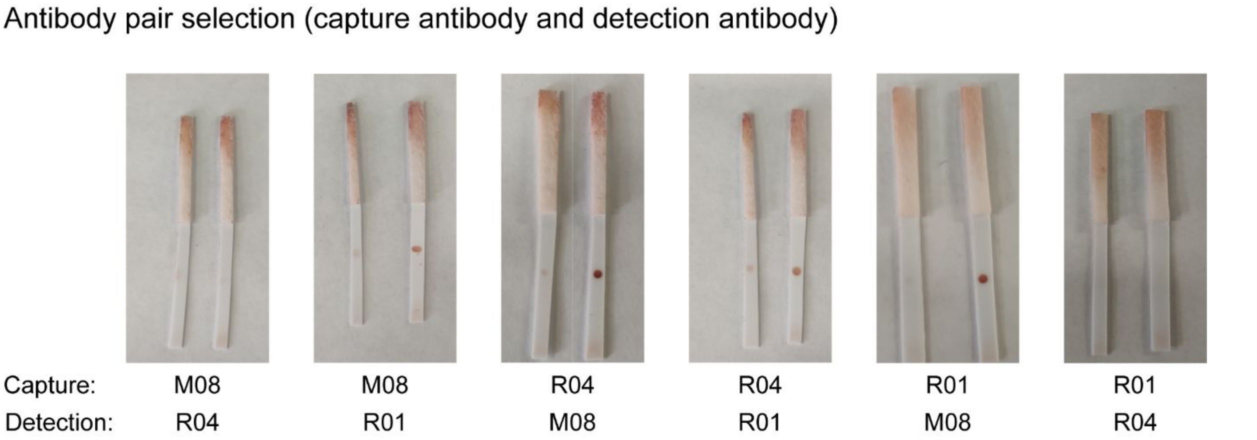

Figure S3. SERS-LFT strips using a dot-blot assay for nucleocapsid protein detection with different antibody combinations. The dots represent detection dots, and no control dots on the strips. The left strip in each photo refers to negative sample while the right strip represents positive sample.

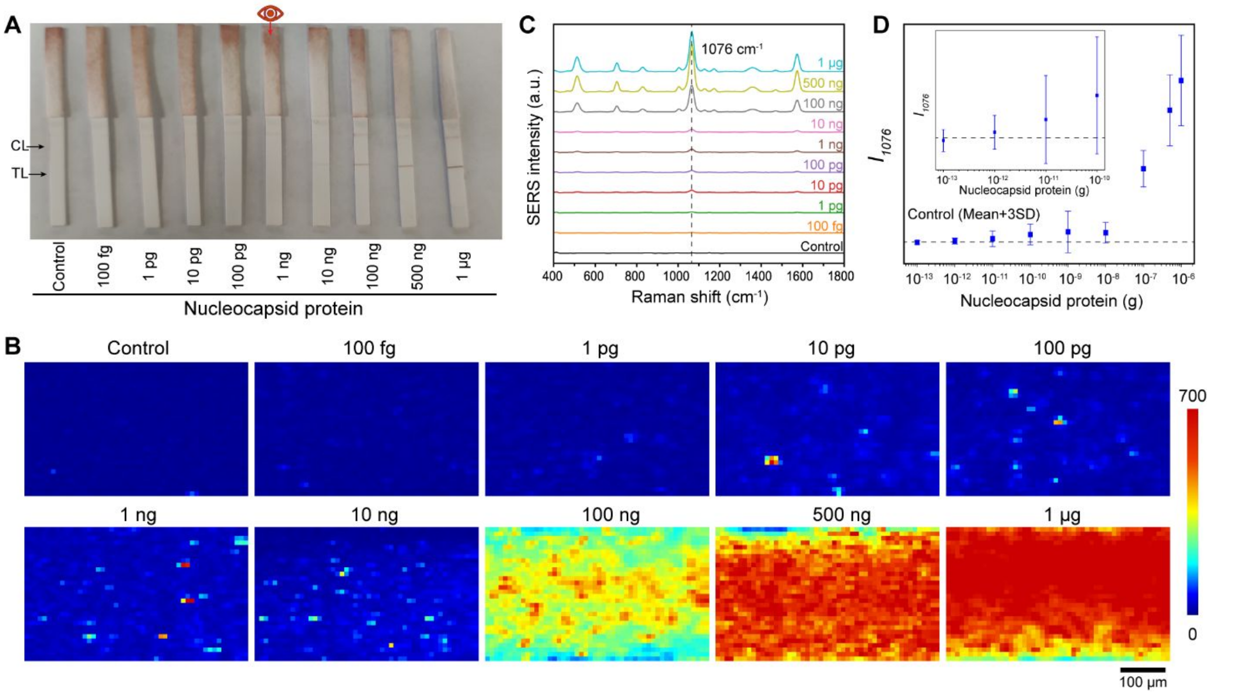

Figure S4. SERS- LFT for SARS-CoV-2 nucleocapsid protein detection. (A) Photograph of the SERS- LFT strips following the detection of nucleocapsid protein at different amounts; (B) SERS maps generated across the scanned area on the test line based on the peak intensity at 1076 cm^{-1} (I_{1076}) for each sample; (C) Average SERS spectra obtained from the scanned area; (D) Comparison of I_{1076} for each sample with control sample. The error bars represent the standard deviations obtained from area scans of each sample.

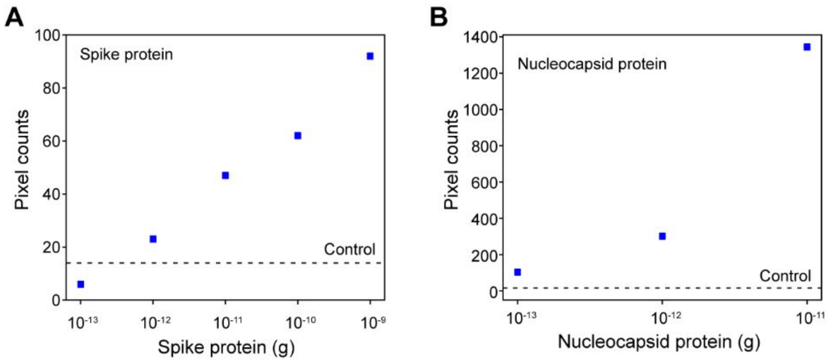

Figure S5. Pixel counts above the threshold for (A) spike protein and (B) nucleocapsid protein at different amounts. The dash lines indicate the pixel counts for control samples.

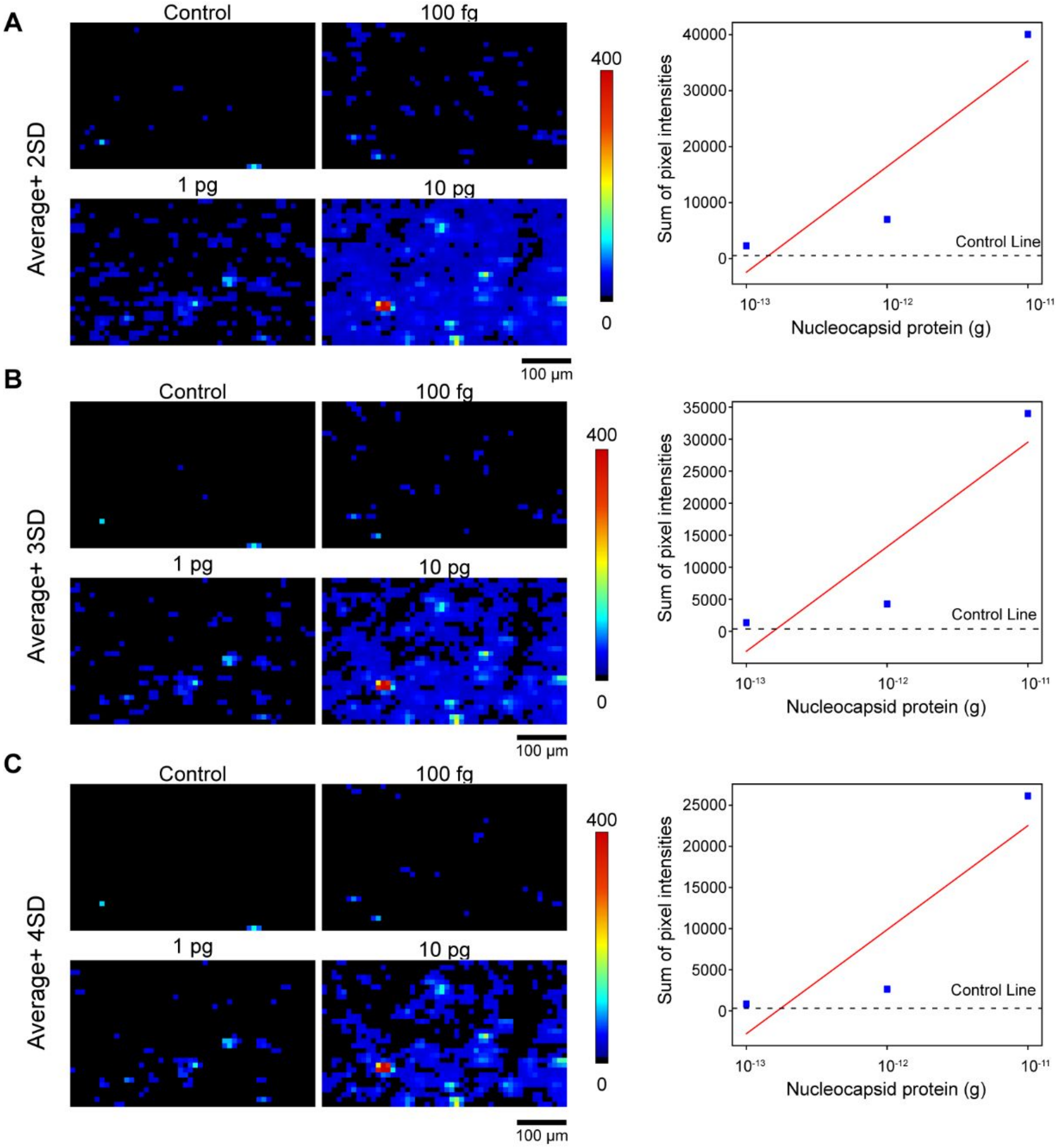

Figure S6. Digital SERS analysis for nucleocapsid protein quantification using different threshold values. (A) Average+2SD; (B) Average+3SD; (C)Average+4SD.

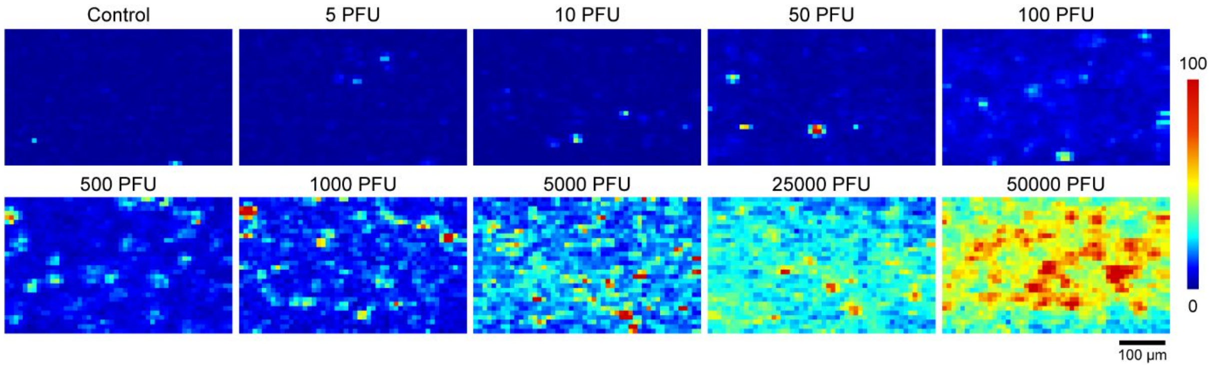

Figure S7. SERS maps generated across the scanned area on the test line for intact SARS-CoV-2 virus detection.

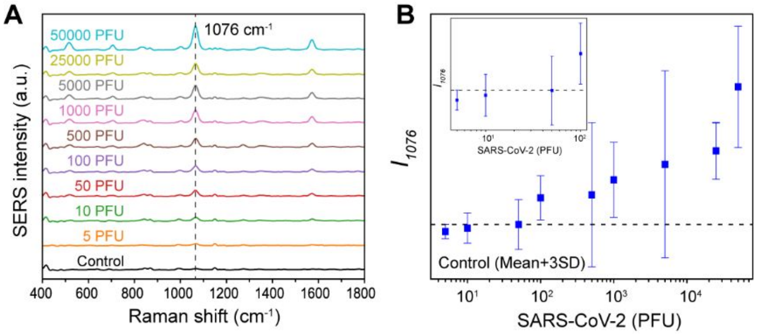

Figure S8. SERS- LFT for intact SARS-CoV-2 virus detection. (A) Average SERS spectra obtained from the scanned area; (B) Comparison of I_{1076} for each sample with control sample. The error bars represent the standard deviations obtained from area scans of each sample.

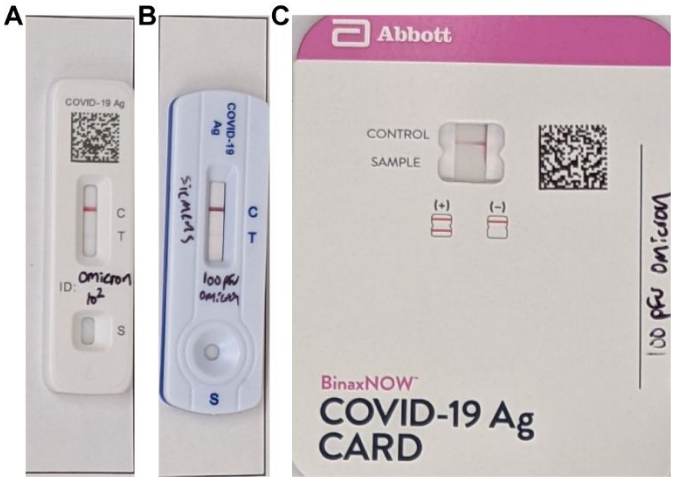

Figure S9. Intact SARS-CoV-2 virus detection using three commercially available antigen test kits at a concentration of 100 PFU. (A) FlowFlex; (B) Siemens; (C) Abbott.

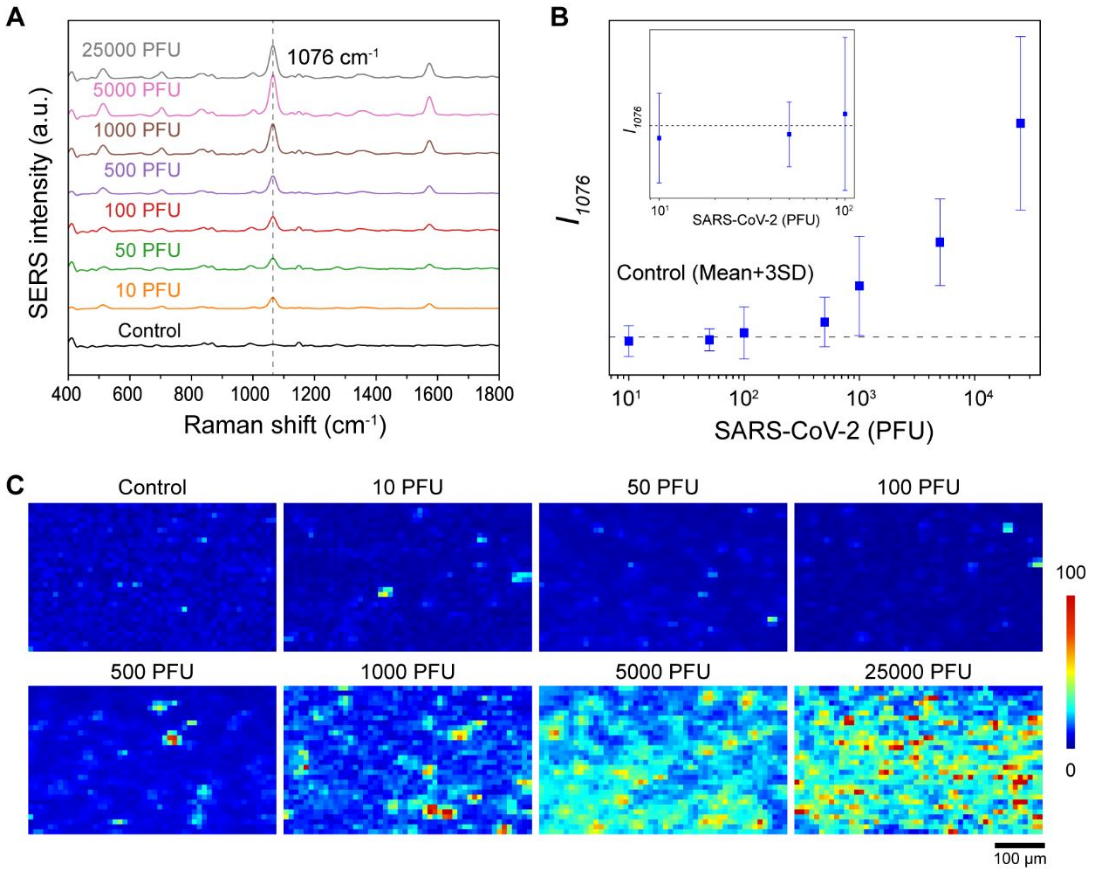

Figure S10. SERS- LFT for intact SARS-CoV-2 detection in indoor dust samples. (A) Average SERS spectra obtained from the scanned area; (B) Comparison of I_{1076} for each sample with control sample. (C) SERS maps generated across the scanned area.

Table S1. Comparison of the detection performance of current digital SERS-based assays for SARS-CoV-2 proteins.

Approach	Target	Matrix	LOD in concentration	Sample volume	LOD in mass	Assay time	Ref.
Digital SERS immunoassay	spike protein	PBS-10	13.7 ng/mL	30 µL	411 pg	5h	1
		saliva	6.3 ng/mL	30 µL	189 pg		
Digital SERS immunoassay	spike protein	/	19 fg/mL	/	/	5h	2
Digital SERS-LFT	spike protein	PBS	18 pg/mL	10 µL	180 fg	30 min	This work
	Nucleocapsid protein	PBS	12 pg/mL	10 µL	120 fg		

References:

(1) Tuckmantel Bido, A.; Brolo, A. G., Digital SERS Protocol Using Au Nanoparticle-Based Extrinsic Raman Labels for the Determination of SARS-CoV-2 Spike Protein in Saliva Samples. *ACS Appl. Nano Mater.* **2023**, *6*, 15426-15436.

(2) Shim, J.-E.; Kim, Y. J.; Choe, J.-H.; Lee, T. G.; You, E.-A., Single-nanoparticle-based digital SERS sensing platform for the accurate quantitative detection of SARS-CoV-2. *ACS Appl. Mater. Interfaces* **2022**, *14*, 38459-38470.
